# Serum concentration of anti-Cytomegalovirus IgG and ischaemic stroke in patients with advanced HIV infection in Malawi

**DOI:** 10.1371/journal.pone.0208040

**Published:** 2018-11-27

**Authors:** Joseph Kamtchum-Tatuene, Zaid Al-Bayati, Henry Charles Mwandumba, Tom Solomon, Stephen E. Christmas, Laura A. Benjamin

**Affiliations:** 1 Institute of Infection and Global Health, University of Liverpool, Liverpool, United Kingdom; 2 Malawi-Liverpool-Wellcome Trust Clinical Research Programme, University of Malawi College of Medicine, Blantyre, Malawi; 3 Department of Clinical Sciences, Liverpool School of Tropical Medicine, Liverpool, United Kingdom; Central University of Tamil Nadu, INDIA

## Abstract

**Background:**

Studies in high-income settings have shown association between Cytomegalovirus (CMV) infection and adverse cardiovascular outcome, especially in HIV infection. We aimed to study the association between serum concentration of anti-CMV IgG and ischaemic stroke in HIV-infected Malawians.

**Methods:**

Our sample was derived from a case-control stroke study in Malawi. Serum concentration of anti-CMV IgG was measured using enzyme-linked immunosorbent assay. Multivariable logistic regression was used to study the association between high concentrations of anti-CMV IgG (above the third tertile) and ischaemic stroke while adjusting for cardiovascular risk factors.

**Results:**

Overall, 139 HIV-positive adults (48.2% women; 48 ischaemic stroke cases and 91 controls; median age: 45 years) were included. The median CD4+ count was 136 and 401 cell/mm^3^ (IQR: [75–278] and [230–533]) in cases and controls, respectively. High concentration of anti-CMV IgG was associated with ischaemic stroke in the univariable model (OR = 2.56 [1.23–5.34]) but not after adjusting for duration of antiretroviral therapy (ART), CD4+ count, and other cardiovascular risk factors (OR = 0.94 [0.29–3.08]). Low CD4+ count was an independent predictor of stroke. There was a negative correlation between serum concentration of anti-CMV IgG and CD4+ count (rho = -0.30, p < 0.001).

**Conclusions:**

High concentration of anti-CMV IgG is not independently associated with ischaemic stroke in HIV-infected Malawians. Larger cohort studies are needed to further investigate the role of humoral response to CMV in the pathophysiology of HIV-associated stroke.

## Introduction

Stroke is a global problem but increasing disproportionately in low-to-middle income countries. This is largely the consequence of lifestyle changes and aging of the population. HIV infection, which is prevalent in sub-Saharan Africa, contributes to young strokes, and further compounds the stroke burden. Early initiation of antiretroviral therapy (ART) has been shown to reduce the overall risk of stroke [1]. However, a higher stroke risk is still observed in immunosuppressed patients within the first 6 months of ART[2] and among those on longer term treatment[3]. In the latter group, the risk is believed to be driven by atherosclerosis while in the former, it is thought to be related to an immune reconstitution-like process[4, 5]. As our knowledge of the mechanism of HIV-related stroke evolves, the contribution of activated inflammatory pathways [6] and chronic infections become more relevant, notably Cytomegalovirus (CMV) infection [7].

In Africa, the prevalence of CMV infection is high, ranging from 81.8% in HIV-negative adults to 94.8% in HIV-positive adults [8]. CMV reactivation reflected by higher CMV-specific T-cell response and increased serum concentration of anti-CMV IgG is associated with surrogate markers of stroke such as carotid intima-media thickness and carotid artery stiffness [7, 9]. A few studies have combined non-AIDS events and cardiovascular mortality and demonstrated an association with CMV infection but no study has focused specifically on a stroke cohort[10, 11]. Furthermore, studies conducted thus far, have been limited to high-income settings where HIV infection is less common and the disease is less advanced [7, 11, 12].

Drugs to prevent CMV reactivation such as Valganciclovir and newer agents such as Letermovir are available and could eventually be offered to reduce the risk of stroke in HIV-infected individuals if the association between stroke and CMV reactivation is demonstrated throughout the different stages of HIV infection and across the spectrum of cardiovascular disease [13–15]. In this study, we sought to determine the relationship between serum concentration of anti-CMV IgG and ischaemic stroke in a group of HIV-infected patients from Malawi.

## Materials and methods

### Study population and ethical considerations

Our sample was derived from a case-control stroke study in Malawi which recruited 222 stroke cases and 503 controls within the community of Blantyre from 2011 to 2012 [2]. Details of the recruitment process have been reported previously [2]. For each participant, one serum sample was prepared from fresh blood collected within 7 days after stroke onset for cases and on the day of enrolment for controls. After the preparation, the serum was aliquoted and stored at −70°C. The research protocols were approved by the Liverpool School of Tropical Medicine and the Malawi College of Medicine Research Ethics Committees. A written informed consent was obtained from each participant. This study consisted of a cross-sectional component restricted to the HIV-infected population in which we examined the relationship between ischaemic stroke and serum concentration of anti-CMV IgG.

### Measurement of serum concentration of anti-CMV IgG by enzyme-linked immunosorbent assay

The concentration of anti-CMV IgG was measured in baseline serum samples using a commercial enzyme-linked immunosorbent assay (ELISA) kit (Genway Biotech Inc., San Diego, USA; catalogue number GWB-892399). The kit has a sensitivity of 100% and a specificity of 97.6%. The ELISA procedure was carried out according to the manufacturer’s instructions and all samples were run in duplicate. The plates were read at 450 nm using an automated reader (Opsys MR, Thermo Fisher Scientific Inc., Waltham, USA) and samples were considered as seronegative if they had a blank-corrected optical density lower or equal to zero. A standard curve was designed for each ELISA plate following the instructions provided with the kit. Intra-assay and inter-assay coefficients of variability (CV) were limited to 10 and 15% respectively. High serum concentration of anti-CMV IgG was defined as a concentration of anti-CMV IgG greater than the third tertile of the distribution in the entire sample.

### Confounders

For each participant, we recorded data on the following risk factors of stroke: age, sex, hypertension (based on blood pressure > 140/90 mmHg or use of antihypertensive drugs), diabetes (based on self-reported physician’s diagnosis, use of antidiabetic drugs or random blood glucose ≥ 11.1 mmol/l), hypercholesterolemia (based on self-reported physician’s diagnosis, use of lipid-lowering drugs or random serum cholesterol level ≥ 6.2 mmol/l), waist-hip ratio (marker of abdominal obesity), CD4+ count (cells/mm^3^), duration of ART, tobacco consumption, alcohol consumption, history of prior transient ischaemic attack (TIA) or stroke, family history of stroke, recent infection defined as fever or treated infection during the previous 2 weeks [16].

Age was dichotomized at the cut-point of 45 years old based on previous findings that the risk of stroke is significantly higher in younger HIV-positive patients [2]. Duration of ART was categorized as no ART, recent ART (< 6 months) or long-standing ART (≥ 6 months). CD4+ count was classified as <350 or ≥350 cells/mm^3^. Alcohol consumption was categorized as current drinker versus no drinker/hardly ever drink. Tobacco consumers were divided into three groups: never smoked, former smoker and current smoker.

Screening tests for infections associated with stroke were performed in stroke cases, but not in controls, as reported previously: *Mycobacterium tuberculosis* infection (MTB), cryptococcal meningitis, *Varicella zoster* infection (VZV) and syphilis [4]. We did not test for other herpesviruses and hepatitis C due to budget limitation.

### Statistical analysis

The relationship between the dichotomized serum concentration of anti-CMV IgG and the stroke risk factors listed above (confounders used in the logistic modelling) was explored using the Chi-square test. The correlation between the serum concentration of anti-CMV IgG and the CD4+ T-cell count was assessed using the Spearman rank correlation test.

Multivariable logistic regression was used to study the association between stroke (dependent variable) and serum concentration of anti-CMV IgG (independent variable) while adjusting for the confounders mentioned above.

Statistical analyses were performed using STATA (version 13; StataCorp LP, USA). All tests were two-tailed and p values < 0.05 were considered to indicate statistical significance.

## Results

### Patients’ clinical characteristics and CMV serology results

**Fig 1:**
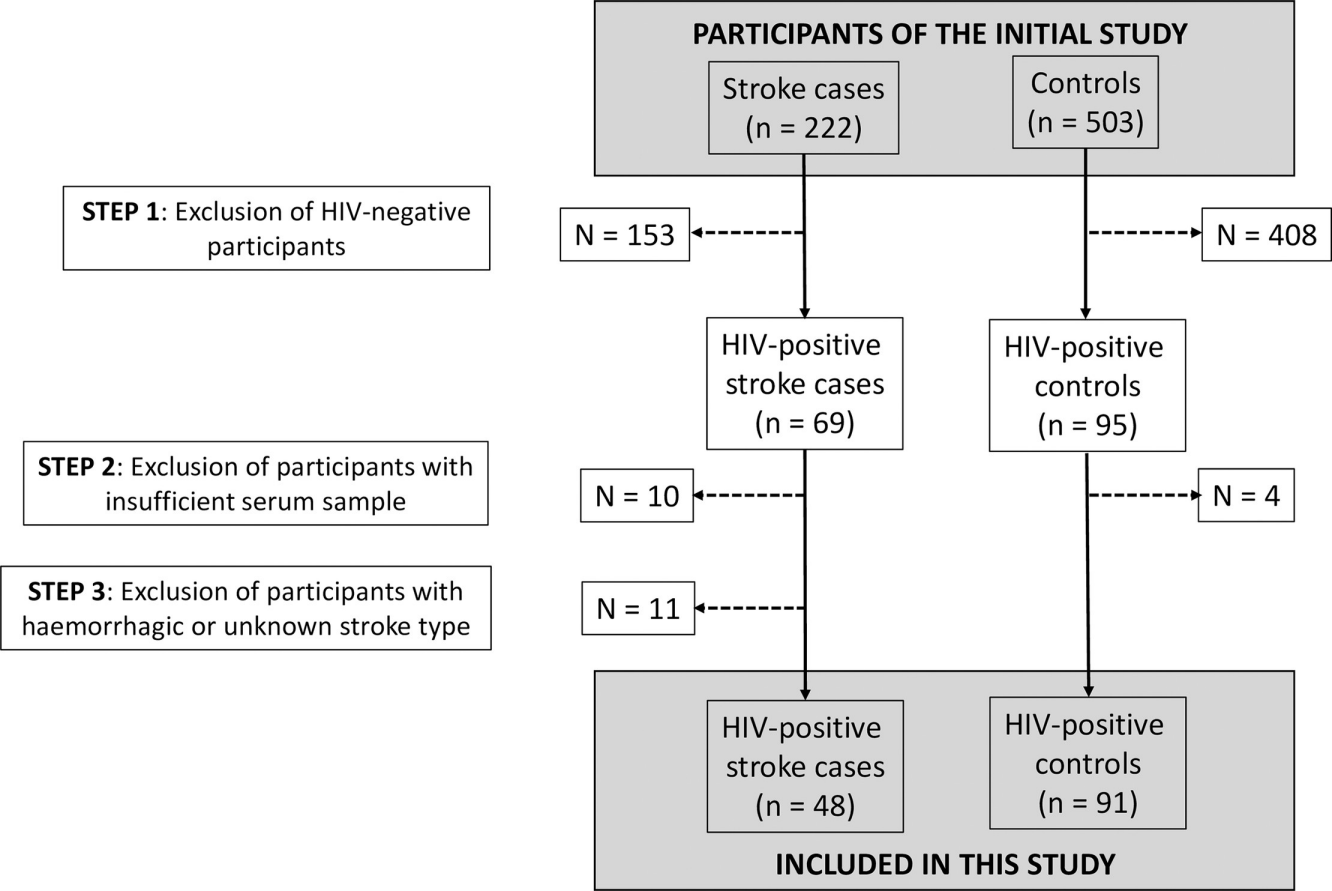
Flow diagram summarizing the participants selection process.

A total of 139 HIV-positive adults (48.2% women) were included in this study, notably 91 controls and 48 ischaemic strokes. The participants selection process is summarized in **Fig 1**. The median age was 39.5 years in the cases and 46.0 years in the controls. The median CD4+ count was 136 and 401 in the cases and the controls respectively (**Table 1**). Nineteen cases (39.6%) and forty-three controls (48.9%) were on ART at the time of inclusion (**Table 1**).

**Table 1:** Baseline characteristics of cases and controls^a^.

Characteristic		Cases (n = 48)	Controls (n = 91)	Total (n = 139)
**Sex**	Female	24 (50.0)	43 (47.3)	67 (48.2)
	Male	24 (50.0)	48 (52.8)	72 (51.8)
**Age (years)**		39.5 (31.0–50.0)	46.0 (37.0–56.0)	45.0 (35.0–54.0)
**Serum concentration of anti-CMV IgG** **(IU/ml)**		18.5 (15.7–23.5)	16.2 (13.5–19.8)	16.9 (14.8–22.4)
**Serum concentration of anti-CMV IgG in patients with values greater than the third tertile (IU/ml)**		24.2 (19.1–29.8)	23.9 (18.9–27.1)	24.0 (18.9–29.8)
**Hypertension**		19 (39.6)	27 (29.7)	46 (33.1)
**Diabetes mellitus**		1 (2.1)	3 (3.3)	4 (2.9)
**Hypercholesterolemia**		7 (14.6)	7 (7.7)	14 (10.1)
**CD4 count (cells/mm^3^)** *(9 missing values)*		136 (75–278)	401 (230–533)	296 (136–460)
**ART duration** ***(3 missing values for controls)***	No treatment	29 (60.4)	45 (51.1)	74 (54.4)
	Recent treatment (< 6 months)	13 (27.1)	7 (8.0)	20 (14.7)
	Long standing treatment (> 6 months)	6 (12.5)	36 (40.9)	42 (30.9)
**Tobacco consumption**	Never smoked	41 (85.4)	72 (79.1)	113 (81.3)
	Former smoker	3 (6.3)	7 (7.7)	10 (7.2)
	Current smoker	4 (8.3)	12 (13.2)	16 (11.5)
**Alcohol consumption** *(1 missing value)*		10 (21.2)	16 (17.6)	26 (18.8)
**Prior TIA or stroke**		8 (16.7)	4 (4.4)	12 (8.6)
**Family history of stroke**		7 (14.6)	16 (17.8)	23 (16.7)
**Recent infection (previous 2 weeks)**		11 (22.9)	14 (15.4)	25 (18.0)
**Waist-hip ratio** *(1 missing values)*		0.9 (0.86–0.94)	0.88 (0.85–0.9)	0.88 (0.87–0.89)

a. Data are reported as count (percentage) for categorical variables and median (interquartile range) for continuous variables.

The prevalence of CMV seropositivity was 100% and anti-CMV IgG concentrations had a Gaussian distribution. The mean intra-assay CV for the anti-CMV IgG concentration was 3.5% (95% CI: 4.3–5.4) while the mean inter-assay CV was 8.0% (95% CI: 3.4–14.7). All patients were asymptomatic for CMV infection.

Sixteen cases of co-infection were identified during the screening [MTB (n = 4), VZV (n = 9), syphilis (n = 3), cryptococcal meningitis (n = 0)] of whom 31.3% (5/16) had a serum anti-CMV IgG concentration greater than the third tertile of the distribution in the study population.

### High serum concentration of anti-CMV IgG and stroke

The serum concentration of anti-CMV IgG was considered high when greater than the third tertile of the distribution in the entire sample (18.94 IU/ml). There were 47 patients (33.8%) with a high serum concentration of anti-CMV IgG including 23 cases and 24 controls. The mean serum concentration of anti-CMV IgG in this subgroup is given in Table 1.

Although high concentration of anti-CMV IgG was significantly associated with stroke in the univariable logistic regression analysis (OR = 2.56; 95% CI: 1.23–5.34), the significance was lost after adjusting for duration of ART, CD4+ count, and other classical risk factors of stroke (adjusted OR = 0.94, 95% CI: 0.29–3.08) (**Table 2**). Low CD4+ count, prior history of cerebrovascular event, and abdominal obesity were independent predictors of stroke (**Table 2**). Using the serum concentration of anti-CMV IgG as a continuous variable in the logistic regression model did not significantly modify the associations reported. There was no interaction between high concentration of anti-CMV IgG and ART status or the CD4+ count.

**Table 2:** Univariable and multivariable logistic regression analyses of factors associated with stroke in HIV positive patients.

Characteristic	Univariable models			Multivariable model		
	OR	95% CI	p	Adjusted OR	95% CI	p
**High concentration of** **anti-CMV IgG**	2.56	1.23–5.34	0.012^a^	0.94	0.29–3.08	0.919
**ART duration**						
*No ART*	1 (ref)			1 (ref)		
*< 6 months*	2.88	1.03–8.08	0.044^a^	2.84	0.74–10.9	0.129
*≥ 6 months*	0.26	0.10–0.69	0.007^a^	0.38	0.89–1.63	0.192
**CD4 count *< 350 cells/mm*^*3*^**	10.88	3.91–30.33	< 0.001^a^	9.82	2.51–38.48	0.001^b^
**Male sex**	0.90	0.45–1.80	0.758	0.95	0.90–10.95	0.934
**Age < 45 years old**	2.54	1.23–5.24	0.011^a^	3.14	0.90–10.95	0.073
**Hypertension**	1.55	0.75–3.23	0.239	2.33	0.70–7.80	0.170
**Diabetes mellitus**	0.62	0.06–6.17	0.687	0.27	0.00–14.76	0.524
**Hypercholesterolemia**	2.05	0.67–6.23	0.206	2.75	0.47–16.2	0.264
**Tobacco consumption**						
*Never smoked*	1 (ref)			1 (ref)		
*Former smoker*	0.75	0.18–3.07	0.692	2.01	0.28–14.6	0.631
*Current smoker*	0.59	0.18–1.93	0.380	0.55	0.07–4.63	0.582
**Alcohol consumption**	1.27	0.52–3.01	0.599	1.00	0.19–5.37	0.996
**Prior TIA or stroke**	4.34	1.24–15.29	0.022^a^	18.12	2.41–135.90	0.005^b^
**Recent infection**	1.63	0.68–3.95	0.274	0.78	0.19–3.20	0.728
**Waist-hip ratio**						
*Tertile 1 (< 0*.*86)*	1 (ref)			1 (ref)		
*Tertile 2 (0*.*86 < value < 0*.*90)*	1.02	0.42–2.46	0.964	1.78	0.49–6.44	0.380
*Tertile 3 (> 0*.*90)*	3.25	1.32–8.06	0.011^a^	4.24	1.10–16.34	0.036^b^
**Prediction characteristics**	-	-	-	AUROC = 0.88, R^2^ = 82.4%		

Statistically significant results are marked as (^a^) in the univariable model and (^b^) in the multivariable model.

### High serum concentration of anti-CMV IgG, immunosuppression and cardiovascular risk factors

**Fig 2:**
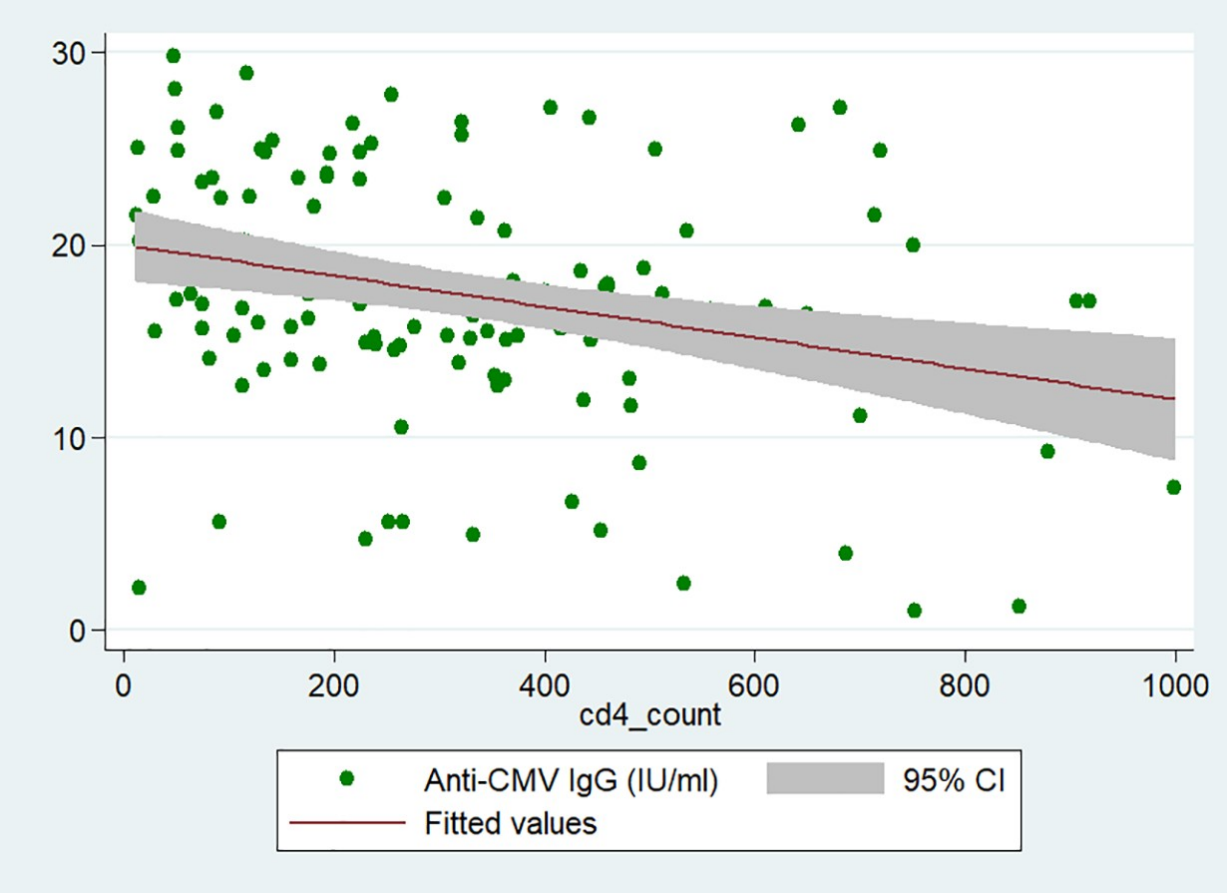
Scatter plot illustrating the negative correlation between anti-CMV IgG concentration and CD4+ count (Pearson’s correlation coefficient = -0.30, p < 0.001; IgG concentration = -8.10^−3^ × CD4 count + 20).

There was a negative correlation between the serum concentration of anti-CMV IgG and the CD4+ count (Spearman rho = -0.30, p < 0.001) as pictured in **Fig 2**. There was no association between high concentration of anti-CMV IgG and the stroke risk factors considered in this study.

## Discussion

We sought to assess the relationship between serum concentration of anti-CMV IgG and stroke in HIV-positive Malawian adults. Our sample included 139 participants (48 ischaemic stroke cases and 91 controls) who were all CMV-positive and represents the largest cohort of HIV-infected stroke patients investigating this question thus far. We found that high concentration of anti-CMV IgG was not an independent risk factor for stroke in contrast to low CD4+ count, prior history of stroke or TIA, and abdominal obesity. A negative correlation was found between the serum concentration of anti-CMV IgG and the CD4+ count (patients with higher concentrations of antibodies had a lower CD4+ count) suggesting that HIV-associated immunosuppression favours CMV reactivation thus leading to a sustained cellular and humoral response that might result in an increased risk of stroke.

There is evidence that high concentration of anti-CMV IgG is a good marker of subclinical CMV reactivation driven by immunosuppression, and correlates well with inflammation, cardiovascular disease and mortality [7, 10, 11, 17, 18]. This is confirmed by the negative correlation between serum concentration of anti-CMV IgG and CD4+ count reported here. Chronic CMV infection causes a dysfunction of the immune system characterized by abnormally high numbers of CMV-specific CD8+ T-cells and a low CD4+/CD8+ T-cell ratio [19]. This perturbation of the immune system is associated with the production of proinflammatory cytokines leading to persistent systemic inflammation, thus increasing non-AIDS morbidity and mortality[19, 20]. Indeed, it remains plausible that the chronic inflammation induced by CMV infection results in endothelial activation, arterial wall remodelling, and a prothrombotic state, and thus predisposes HIV-infected patients to long term cardiac and cerebral vascular risk [9, 11, 13].

The negative impact of CMV reactivation on the immune system can also impair the host’s ability to mount a balanced reconstitution of the immune system following ART initiation [21]. This could increase the risk of developing an immune reconstitution inflammatory syndrome (IRIS)–driven by CMV itself or other pathogens–during the early phase of ART. The occurrence of an IRIS would further increase the risk of stroke by aggravating the systemic prothrombotic state or by promoting local thromboembolic processes in the case of CMV-related vasculitis [13]. Although the association between stroke and recent initiation of ART was lost in the multivariable analysis, we cannot completely rule out the contribution of CMV reactivation to the pathophysiology of stroke in participants that developed stroke within 6 months of ART initiation [2].

Our study was not large enough to confidently prove an independent association between CMV reactivation and stroke. While the pathophysiological hypotheses discussed above are biologically plausible, we were unable to confirm whether CMV reactivation is a mediator on the pathway from HIV infection to stroke or an innocent bystander. However, there is evidence to suggest that immune response to CMV rather than the direct effect of viral replication is responsible for the adverse clinical outcome seen in many studies [12]. Therefore, the recently launched WHO test-and-treat policy could decrease the incidence of CMV reactivation by reducing the degree of pre-ART immunosuppression [22]. This could translate into a reduction in the burden of adverse cardiovascular outcomes in HIV-infected individuals. In Sub-Saharan Africa where the greatest burden of HIV infection is found [23], a lot remains to be done to reduce the proportion of HIV-infected individuals who present late for diagnosis and treatment [24]. Until this is achieved, the association between CMV reactivation and immunosuppression could pose an additional cardiovascular risk in vulnerable HIV-infected populations.

This is the first study to explore the relationship between stroke and serum concentration of anti-CMV IgG in HIV-infected patients, in an African setting (Malawi). However, the small sample size provided insufficient power to detect an independent association between high concentration of anti-CMV IgG or recent ART initiation and stroke. Moreover, our estimation of the serum concentration of anti-CMV antibodies could be improved by multiple sampling. We also acknowledge that more specific methods to assess CMV reactivation (e.g. plasma CMV DNA load and CMV-specific T-cell response) [25–27] could have been used but the retrospective analysis of our samples limited our options. Furthermore, we did not measure the total IgG concentration in this study and therefore, cannot fully rule out the possibility of high anti-CMV IgG being the consequence of a non-specific hyper-immunoglobulinemia. Finally, the absence of CMV-negative subjects prevents comparison of ART effect and stroke risk between CMV-positive and CMV-negative patients living with HIV and therefore limits the possibility to infer causality when examining the complex relationship between CMV infection, ART and stroke. However, this limitation is difficult to overcome given that CMV infection is almost ubiquitous in HIV-endemic low-income settings [28].

In conclusion, this study suggests that, in individuals with advanced HIV infection, high serum concentration of anti-CMV IgG is not independently associated with stroke. However, it is associated with immunosuppression, which is an independent risk factor for stroke, and thus might have a mediating role. Further cohort studies with larger sample size are needed to clarify the contribution of CMV reactivation to the short- and long-term risk of stroke in HIV-infected individuals before advocating the use of specific anti-CMV drugs for primary prevention. Such studies should combine rigorous clinical and CMV-specific laboratory tools for accurate diagnosis of CMV reactivation and assess the contribution of other herpesviruses to the chronic inflammation observed in HIV-infected individuals. They should also aim to include a greater number of patients initiating ART (representing only 14.7% of our sample) to test the hypothesis that the potential mediating role of CMV reactivation in the pathophysiology of HIV-associated stroke is important at the time of immune reconstitution in people with low baseline CD4+ counts.

## Supporting information

S1 Appendix Study dataset. (DTA)
